# Residual rotation of forearm amputation: cadaveric study

**DOI:** 10.1186/s12891-020-3050-x

**Published:** 2020-01-18

**Authors:** Geon Lee, Sung-Jae Kim, Joo Hyung Ha, Chang-Hun Lee, Young Jin Choi, Kwang-Hyun Lee

**Affiliations:** 1 Department of Electrical and Electronic Engineering, Hanyang University, Ansan, South Korea; 2 Department of Orthopaedic Surgery, Hallym University College of Medicine, Hwasung, South Korea; 3 Department of Orthopaedic Surgery, Eulji Medical Center, Eulji University College of Medicine, Seoul, South Korea; 4 Department of Orthopaedic Surgery, Hanyang University College of Medicine, 222 Wangsimni-ro, Seongdong-gu, Seoul, 04763 South Korea

**Keywords:** Rotation, Forearm amputation

## Abstract

**Background:**

The purpose of this study was to investigate residual rotation of patients with forearm amputation and the contribution of involved muscle to residual rotation.

**Methods:**

Testing was performed using five fresh-frozen cadaveric specimens prepared by isolating muscles involved in forearm rotation. Amputation was implemented at 25 cm (wrist disarticulation), 18 cm, or 10 cm from the tip of olecranon. Supination and pronation in the amputation stump were simulated with traction of involved muscle (supinator, biceps brachii, pronator teres, pronator quadratus) using an electric actuator. The degree of rotation was examined at 30°, 60°, 90°, and 120° in flexion of elbow.

**Results:**

Average rotation of 25 cm forearm stump was 148° (SD: 23.1). The rotation was decreased to 117.5° (SD: 26.6) at 18 cm forearm stump. It was further decreased to 63° (SD 31.5) at 10 cm forearm stump. Tendency of disorganized rotation was observed in close proximity of the amputation site to the elbow. Full residual pronation was achieved with traction of each pronator teres and pronator quadratus. Although traction of supinator could implement residual supination, the contribution of biceps brachii ranged from 4 to 88% according to the degree of flexion.

**Conclusions:**

Close proximity of the amputation site to the elbow decreased the residual rotation significantly compared to residual rotation of wrist disarticulation. The preservation of pronosupination was 80% at 18 cm forearm stump. Although the pronator teres and the pronator quadratus could make a full residual pronation separately, the supinator was essential to a residual supination.

## Background

Amputation is a final option to treat problem in the extremities due to trauma, malignant neoplasm, infection, or vascular disease [1–5]. Since the function of amputated extremity is diminished after amputation, the usage of prosthesis is needed. However, up to date, the function of prosthesis is primitive due to focus on aesthetic point rather than function. In addition, the prosthesis was attached to the stump with self-suspended socket. For this reason, the residual rotation of forearm has not been of interest to surgeons [6]. Surgeons have tried to preserve the affected limb as long as possible in amputation surgery for patients to use the socket prosthesis.

On the other hand, technology of robot engineering is changing. Major amputation of the upper extremity is now taking a new turn through advances in robotic hands that can replicate human’s hand gradually with light weight [7, 8]. Some trials have already been conducted to apply robotic hands to forearm amputation [9, 10]. Furthermore, osseointegration has also been applied to patients with trans-radial amputation [11]. Osseointegration which anchors a prosthetic device directly to the skeleton could allow natural rotation of the transradial amputee. These changes demand more precise knowledge about the residual rotation of the forearm along the amputation level.

The purpose of this study was to investigate residual rotation of patients with forearm amputation along the amputation level and the contribution of involved muscles to residual rotation.

## Methods

### Specimen preparation

**Fig. 1:**
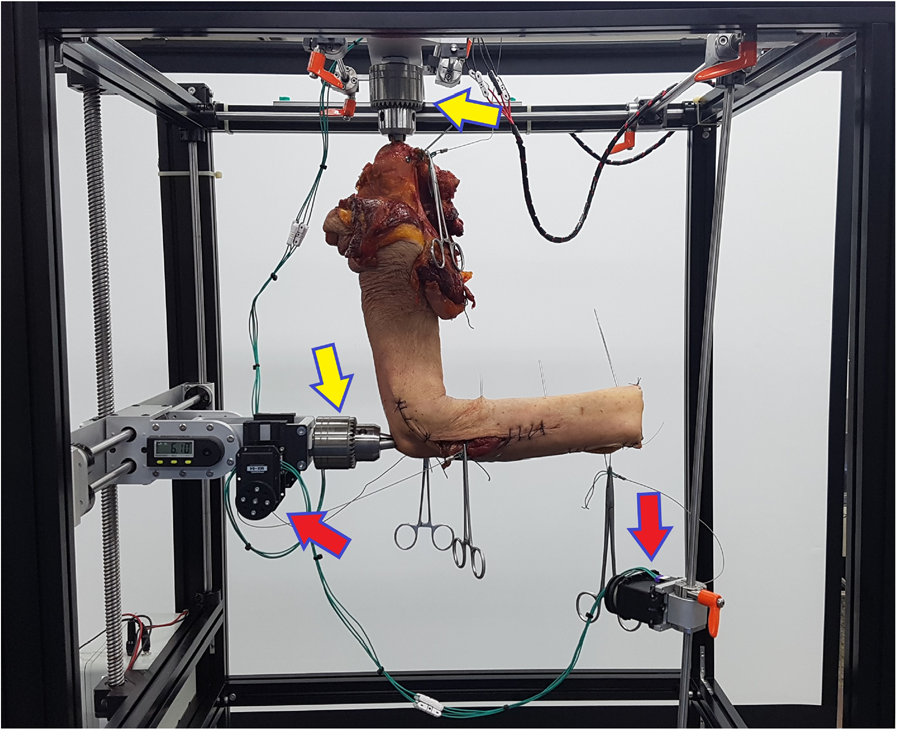
A custom forearm motion simulator system including two dynamic drill chucks (yellow arrows) and four electric actuators (red arrows)

Testing was performed on five fresh-frozen cadaveric specimens (mean age, 72.5 years; range, 47–88 years; five men) without history of trauma or surgery of the upper extremity. These cadavers used in this study were provided by the College of Medicine, Hanyang University. Fresh frozen cadavers were thawed at room temperature for 24 h. These upper extremity specimens were separated from the torso at the level of glenohumeral joint with scalpel. Biceps brachii (BIC), supinator (SUP), pronator teres (PT), and pronator quadratus (PQ) were isolated with fine dissection. Origins of these muscles were detached and sutured using number 5 Ethibond (Ethicon, Somerville, NJ, USA) with Krackow method. Suture materials were attached to individual electric actuator that was placed on the physiologic line of action of each muscle (Fig. 1). Additionally, to maintain the muscle moment arms physiologically as much as possible, soft tissue and skin were kept intact.

### Testing apparatus

The principle function of the testing apparatus was to provide independent load control for the tendon and motion of the cadaveric forearm. A custom forearm motion simulator system including two dynamic drill chucks and four electric actuators (MX-64r, Robotis, Korea) was developed for the test. Intramedullary nails (Acumed, Hilsoboro, OR, USA) were inserted into the humerus and ulna. Proximal ends of nails protruded to dock into dynamic drill chucks. A dynamic drill chuck was placed in the roof of the testing apparatus for fixation of humerus. The other dynamic drill chuck was placed with a digital protractor on the side of the apparatus. The proximal end of the ulnar nail was secured. Specimens could change a position from 30° to 120° in flexion of elbow.

### Simulation of motion

We first tested passive motion by manually rotating the forearm through a full arc of motion from pronation to supination. An iterative loading protocol was used for the supinator muscle of each specimen to determine the minimum load necessary to produce quasi-static supination motion of the forearm with the humerus oriented vertically. Magnitudes of biceps, supinator, pronator teres, and pronator quadratus loads were derived by apportioning muscle loading. The ratio of muscle loading was determined based on previous studies of forearm muscle electromyographic activity and physiologic muscle cross-sectional area [12, 13]. BIC and SUP were loaded to overcome a 20-N counterforce from early initiation of the pronator teres, ensuring that supination began in a fully pronated position and generated simulated active supination at a rate of 5 mm/s. PQ and PT were then loaded to overcome a counter force of supinator vice versa. Simultaneous electric actuator loads were regulated with proportional pressure controllers (CM 7000, Robotis, Korea) under computer control using a custom-programmed software.

### Amputation and measurement

Taylor CL has reported residual rotation of forearm amputation at four different level: 4 in., 6 in., 8 in., and 10 in. from the lateral condyle of the humerus [14]. The level of amputation was determined according to Taylor CL [14] . Correction was performed considering the size of Korean male cadavers. Distal radio-ulnar joint and interosseous membrane are static stabilizers of forearm rotation [15]. We tried to simulate patients with intact distal radioulnar joint and interosseous membrane, patients with preserved interosseous membrane including central band and proximal membranous portion, and patients who just preserved proximal portion of interosseous membrane. Thus, amputation of 8 in. from the lateral condyle of humerus was dropped out. Amputation was made at three points: 25 cm (wrist disarticulation), 18 cm, and 10 cm from the tip of olecranon. Skin incisions were designed with equal-length flaps along volar and dorsal aspects of the forearm. Osteotomies in the radius and ulna were made 1 cm proximal to the level of the skin incision. Skin and wound were closed in layers after amputation. 3 K-wires with diameter of 2.0 mm were placed into the radius of neutral position just proximal to predetermined amputation level before amputation. Residual rotation was measured with a goniometer for each of five cadavers after the amputation at three levels respectively. The measurement was repeated at 30°, 60°, 90°, and 120° in flexion of elbow.

Supination testing was performed in the following order: isolated supinator loading, isolated biceps loading, and simultaneous both muscle loading. Pronation testing was executed in the following order: isolated pronator quadratus loading, isolated pronator teres loading, and simultaneous both muscle loading in the same manner. Repeatability was determined from five successive trials. The mean was employed as a measurement variable.

### Statistical analysis

The mean of five trials was calculated and employed for statistical analysis. Residual rotation at different levels of amputation and effect of elbow flexion on residual rotation were analyzed with Kruskal Wallis test. Mann Whitney U test was used for subgroup analysis. A *P* value of less than 0.05 was regarded as statistically significant.

## Results

**Fig. 2:**
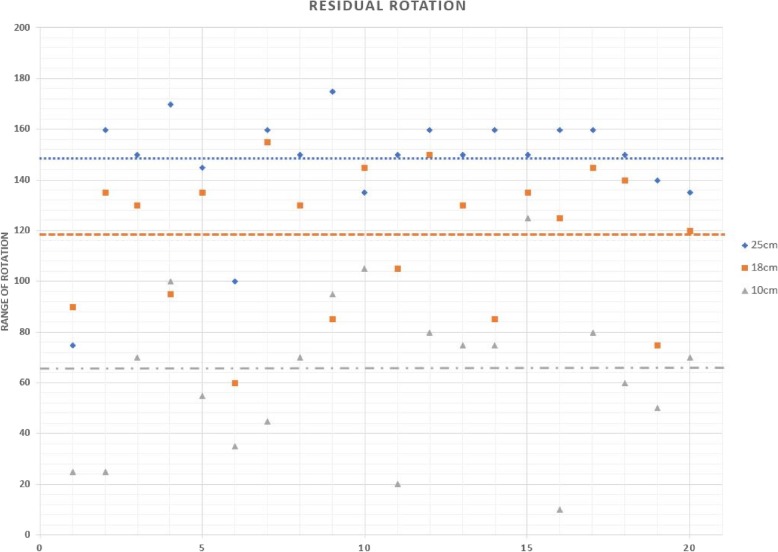
A scatter chart showing the mean residual rotation after forearm amputation. In x axis, number 1–5 was the measurement of 120° of elbow. Number 6–10 was the measurement of 90° of elbow. Number 11–15 was the measurement of 60° of elbow. Number 16–20 was the measurement of 30° of elbow

**Fig. 3:**
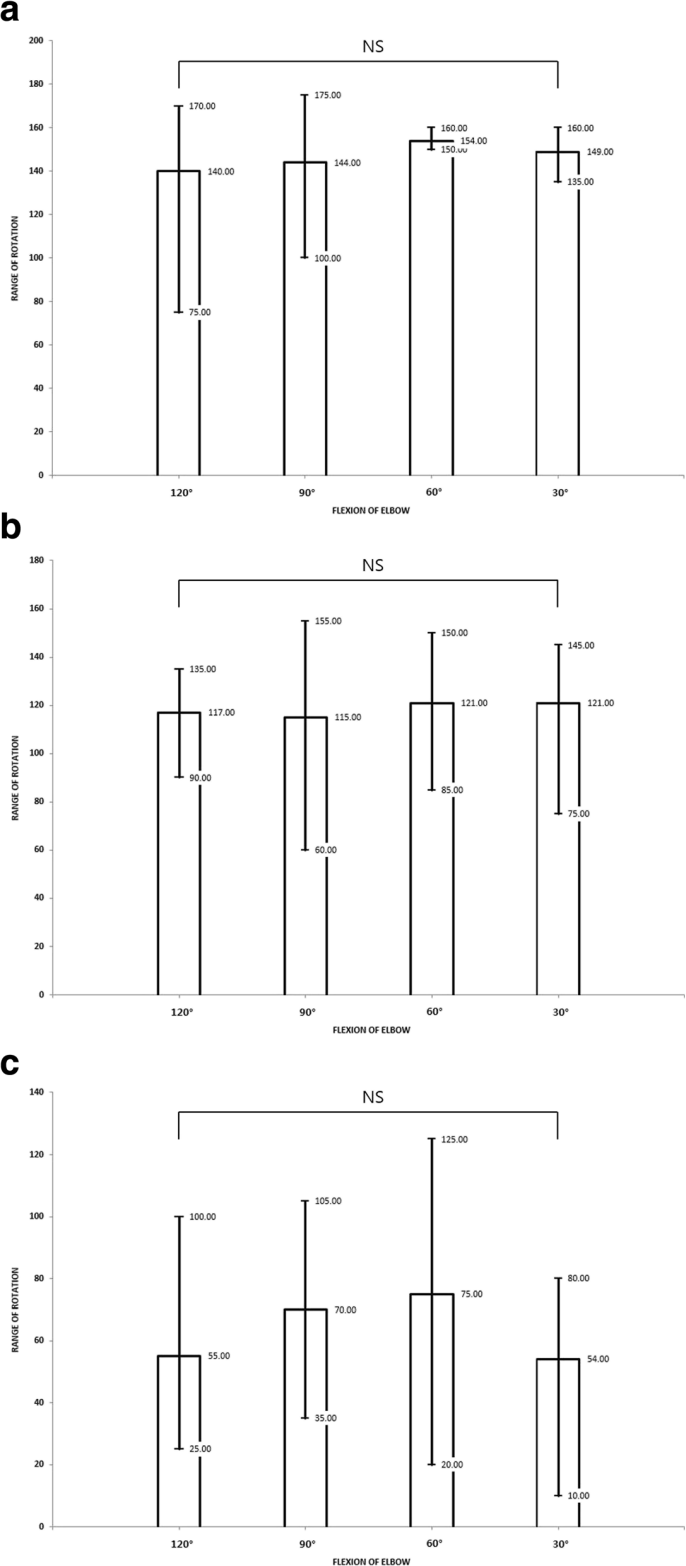
Charts showing the effect of elbow flexion on redisual rotation. ⓐ 25 cm forearm stump, ⓑ 18 cm forearm stump, ⓒ 10 cm forearm stump, NS: not significant

The average load to supinator was 22.4 N (range, 20~25 N). Average loads for biceps, pronator teres, and pronator quadratus were 45.8 N (range, 41~51 N), 22.4 N (range, 20~25 N), and 16.8 N (range, 15~19 N), respectively. When amputation was conducted at the level of 10 cm from the olecranon, the insertion of pronator teres was partially transected in 4 of 5 cadaveric specimens. The remnant of the insertion was detached during the simulation of forearm rotation. The average rotation of 25 cm forearm stump was 148° (SD: 23.1). The rotation was significantly decreased to 117.5° (SD: 26.6) at 18 cm forearm stump (*p* < 0.01) and 63° (SD: 31.5) at 10 cm forearm stump (*p* < 0.01, Fig. 2). The average pronation was 73.4° (SD: 13.5) at 25 cm stump, 53.8° (SD: 20.5) at 18 cm stump, and 8.8° (SD: 4.8) at 10 cm stump. The average supination was 74° (SD: 13.5) at 25 cm stump, 64.8° (SD: 28) at 18 cm stump, and 61.8° (SD: 33.1) at 10 cm stump. The effect of elbow flexion on residual rotation was not statistically significant (*p* > 0.05, Fig. 3).

**Fig. 4:**
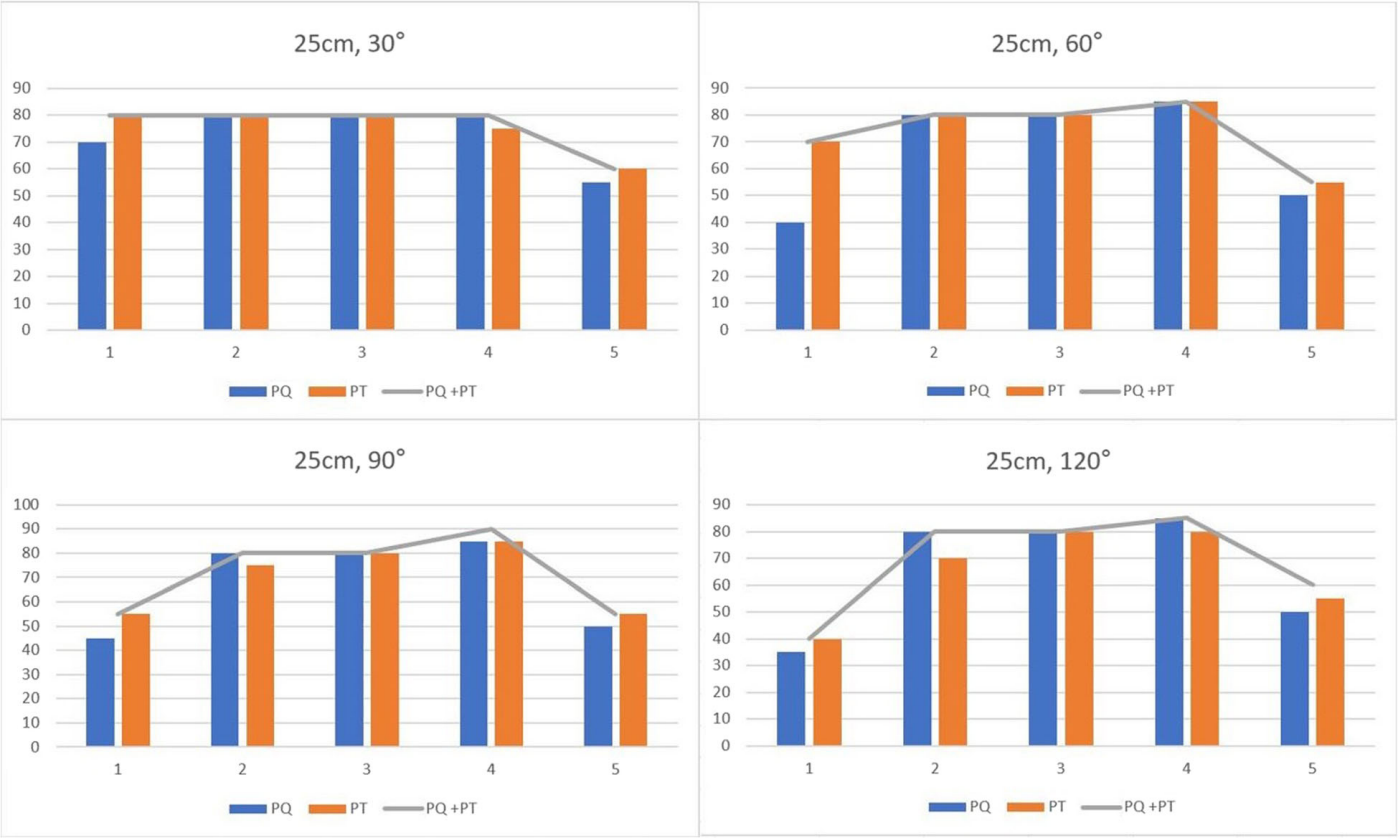
Charts showing residual pronation with separate fraction of PT (pronator teres) and PQ (pronator quadratus) in 25 cm forearm stump

**Fig. 5:**
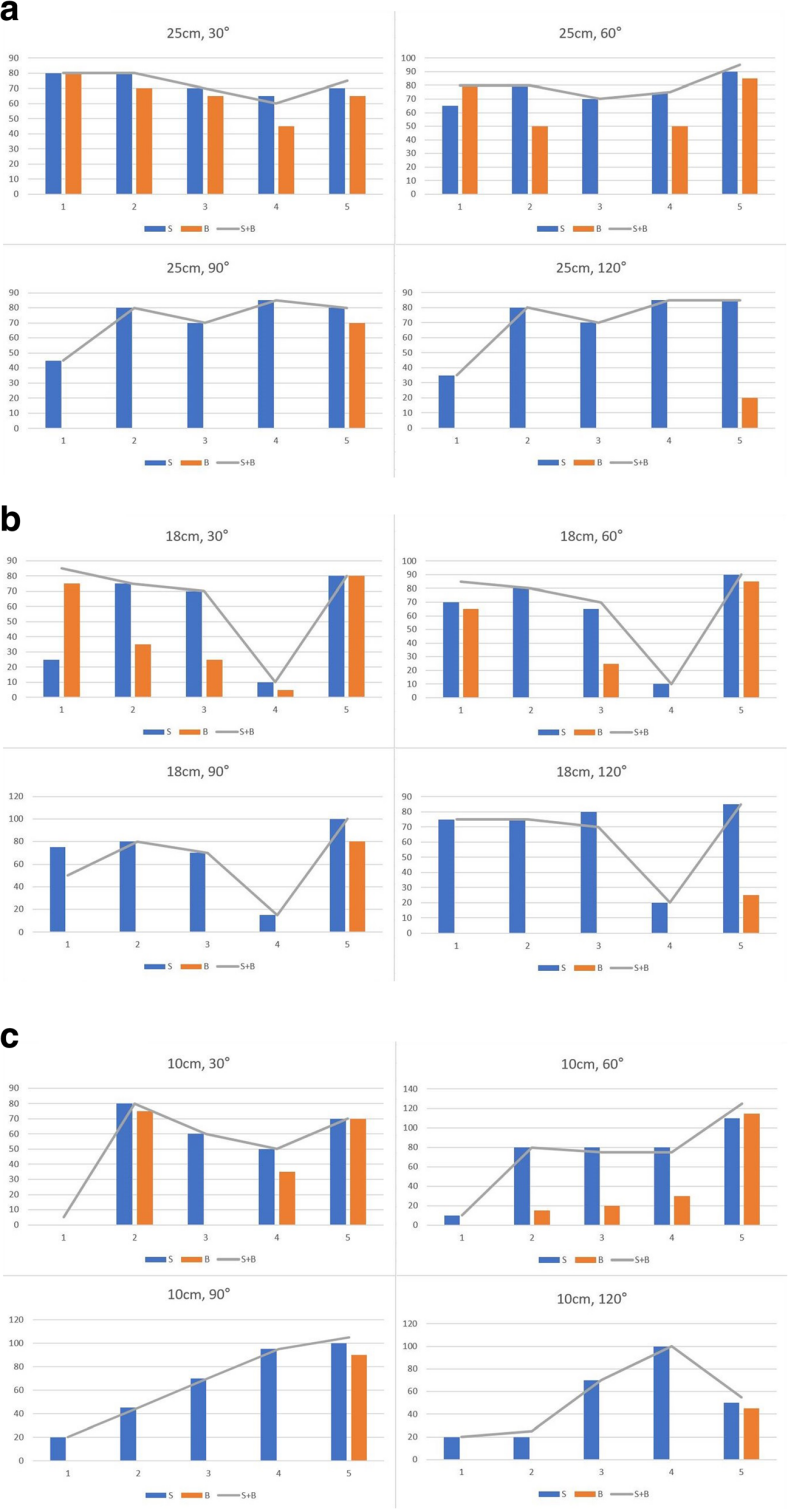
Charts showing residual supination with separate fraction of S (supinator) and B (biceps brachii). ⓐ 25 cm forearm stump, ⓑ 18 cm forearm stump, ⓒ 10 cm forearm stump

Separate traction of pronator teres and pronator quadratus made the pronation close to the full residual pronation, achieving simultaneous traction of pronator teres and pronator quadratus (Fig. 4). The pronation at 18 cm or 10 cm stump was only made by the traction of PT due to deficiency of PQ. Although traction of supinator could make residual supination, contribution of biceps brachii was inconsistent (Fig. 5). Traction of biceps brachii in the elbow of 90 degrees and 120 degrees failed to materialize significant supination. Traction of biceps brachii resulted in a minimum average value of 4% of residual supination at flexion of 120 degrees in 25 cm stump and a maximum average value of 88% of residual supination at flexion of 30 degrees in 25 cm stump.

## Discussion

Amputation is not preferred by a surgeon because the nature of the surgery is not to recover function, but to eliminate function and humanity. Taylor CL investigated socket rotation based on radioulnar rotation of six amputees in 1954 [14]. Since then, there has been no follow-up trial to confirm their results. Although a surgeon could manage forearm amputation for decades through insightful investigation, residual rotation of forearm amputation has been out-of-interest because residual rotation of forearm amputation is useless with conventional socket prosthesis. However, osseointegration in the transradial amputee can restore the natural forearm rotation now [11]. In the near future, reconstructive surgery with biomimetic hands will become possible. Thus, there is a need to address residual rotation of forearm amputation.

In wrist disarticulation, rotation axis can be preserved through intact distal radioulnar joint. Complete rotation was observed even without hands. In the 18 cm forearm stump, the preservation of pronosupination was 80% compared to residual rotation of wrist disarticulation. Although supinator, biceps brachii, and pronator teres were preserved, loss of distal radioulnar joint derailed the balanced rotation and decreased the residual rotation. In the 10 cm forearm stump, residual rotation fell below half of the residual rotation of wrist disarticulation. Insertions of pronator teres were partially transected in 4 of 5 cadaveric specimen of the 10 cm forearm stump. The majority of interosseous membranes (central band and distal oblique bundle) were also resected in all specimens. The pronation simulation test at the 10 cm stump could be completely finished in only one cadaver. The residual pronation was smaller than the residual supination in the 10 cm forearm stump. Such smaller pronation ability could be partially explained by diverging loads applied to the radius by supinator muscle and the biceps brachii which might have aggravated the unstable rotation without distal radioulnar joint or interosseous membrane [16].

Close proximity of the amputation site to the elbow significantly decreased residual rotation. While Taylor CL investigated the socket rotation of amputees, we measured the rotation of radius around the fixed ulna in fresh-frozen cadavers. Although direct comparison between Taylor’s study and our study was not possible, our finding was consistent with the decreasing tendency of rotation as the level of amputation got shorter. Moritomo H et al. [17] have reported that distal three ligaments of the interosseous membrane are essentially isometric stabilizers of the forearm. Two proximal ligaments (proximal oblique cord and dorsal oblique accessory cord) changed substantially in length, with their attachments out of the course of the axis. In the 25 cm forearm amputation, distal radio-ulnar joint (DRUJ) and all contents of interosseous membrane were intact. In the 18 cm forearm amputation, DRUJ and distal membranous portion including distal oblique bundle were eliminated while proximal membranous portion and middle ligamentous complex remained intact. In the 10 cm forearm amputation, DRUJ, distal membranous portion, and middle ligamentous complex were all eliminated while only fluctuating proximal membranous portion remained. Unstable and short forearm stump resulted in a decrease of residual rotation.

Separate traction of pronator quadratus and pronator teres could make an equal pronation to residual pronation resulting from simultaneous traction of both muscles in specimens of 25 cm amputation. No study has shown that solitary contraction of pronator quadratus can make a pronation of forearm. However, many clinical studies have investigated pronation with or without repair of pronator quadratus in patients treated surgically for distal radial fractures and shown that there is no significant difference in the range of motion between the two groups (pronation with or without repair of pronator quadratus) [18–20]. Our results support these findings, showing that independent traction of pronator teres could make an equal pronation to residual pronation resulting from simultaneous traction of both muscles in the specimens of 25 cm amputation.

Another finding of our study was that supination made by independent traction of biceps brachii was affected by flexion of elbow. While biceps brachii functioned primarily as a powerful supinator of the forearm when the elbow partially flexed from 30° to 60°, the biceps brachii did not function primarily in 90° or 120°. Considering that the main action of biceps brachii is supination and flexion of elbow joint, supination of biceps brachii can be synergistic when it is combined with the elbow flexion. Castration of flexion force due to fixation of the ulna in 90° and 120° of elbow might have decreased the supinator effect of biceps brachii. However, traction of supinator made a supination equal to residual supination with simultaneous traction of both muscles. These results were consistent with previous studies, demonstrating the importance of the supinator muscle in forearm supination. In electromyographic studies of forearm supination, the supinator muscle was the most active one in unresisted supination, showing increased biceps activity with heavy loading [21]. Selective denervation of the supinator muscle by peripheral blockade of the radial nerve with preserved biceps function via the musculocutaneous nerve has been shown to decrease the supination strength by 64% [22]. These findings might support previous studies that compared clinical results of biceps tenotomy with that of tenodesis. These studies have shown that there is no significant difference in supination power between the two groups [23, 24].

Our results revealed that the effect of elbow flexion on residual rotation was not statistically significant. Independent traction of pronator teres and pronator quadratus can make full residual pronation at any position of the elbow. Although the attribution of biceps brachii to supination was influenced by the flexion of elbow, supination caused by the traction of supinator was constant.

Our study has several limitations. First, muscle contraction was substituted with traction of muscle. It is an inherent limitation of a cadaveric study. We could not guarantee the load to pull the muscle completely for forearm rotation. Second, maximum loads applied to PQ, PT, and biceps brachii were chosen based on derived ratios. However, these ratios could only provide an estimate of the relative force produced by muscles, not the absolute force. Further study is needed to determine effects of alternative muscle-loading. Third, the accuracy in modeling muscles using a single suture line, especially those muscles with a broad origin (such as PQ, PT, and supinator muscles), might be questionable. Fourth, the effect of myodesis using forearm muscle on radius or ulna was not evaluated. The scar tissue is often present abundantly in the stump. Remained muscles and scarred soft tissue could influence the residual rotation. Finally, our study was conducted with Korean male cadavers. We corrected the level of amputation considering the size of Korean male cadavers. Thus, results of this study could not be applied to the general population due to differences in race and/or gender.

## Conclusions

This cadaveric study investigated residual rotation of forearm amputation. Our findings showed that close proximity of the amputation site to the elbow significantly decreased residual rotation. We also found that the rotation became more unstable in shorter stump. The contribution of involved muscle to residual rotation can help us understand residual rotation of the forearm. To restore natural rotation of forearm amputation, at least the insertion of pronator teres should be preserved.

## Data Availability

The datasets used and/or analysed during the current study are available from the corresponding author on reasonable request.

## Abbreviations

BIC: Biceps brachii
DRUJ: Distal radio-ulnar joint
PQ: Pronator quadratus
PT: Pronator teres
SUP: Supinator
